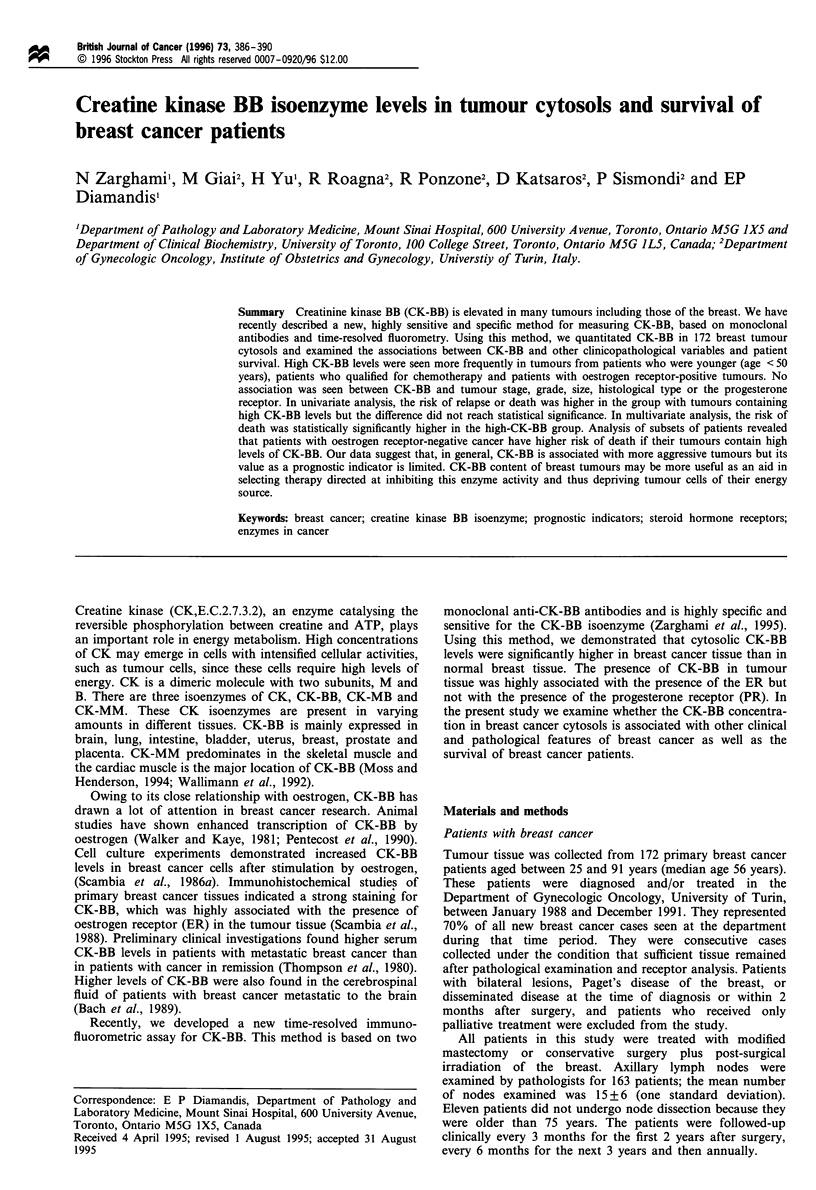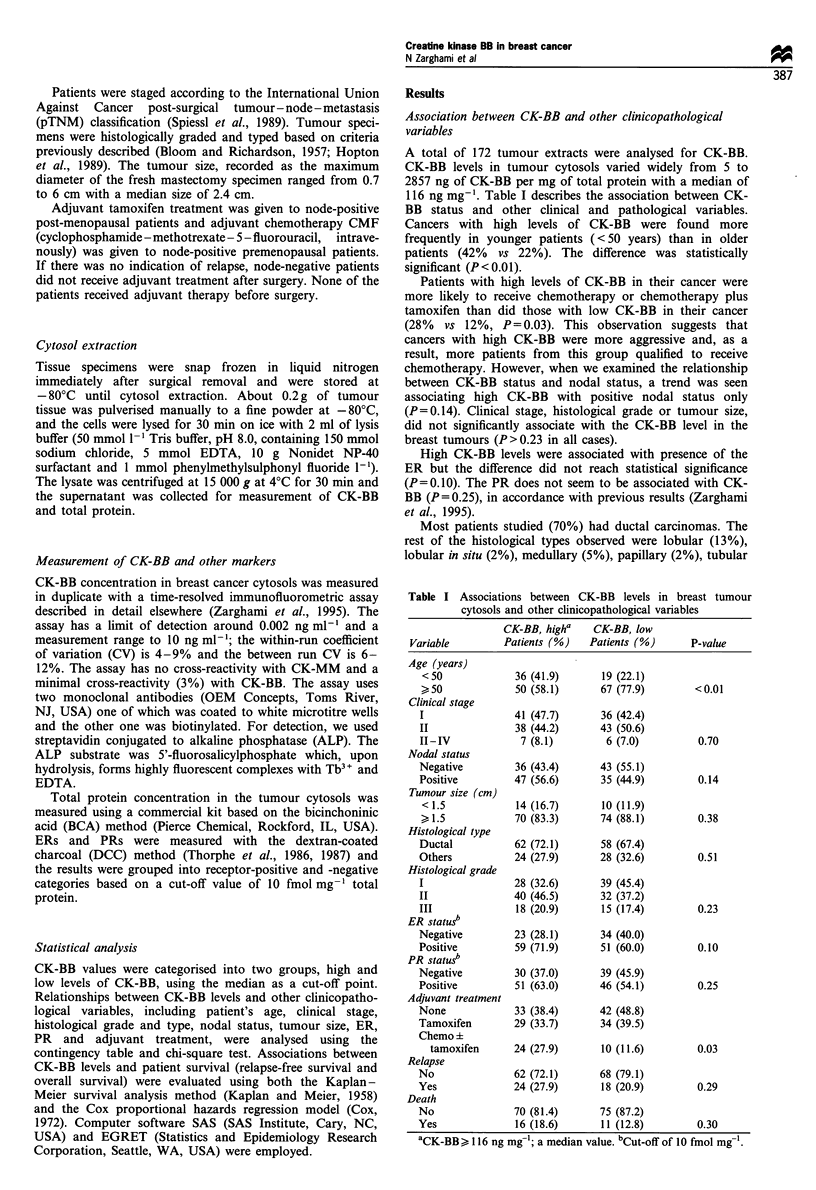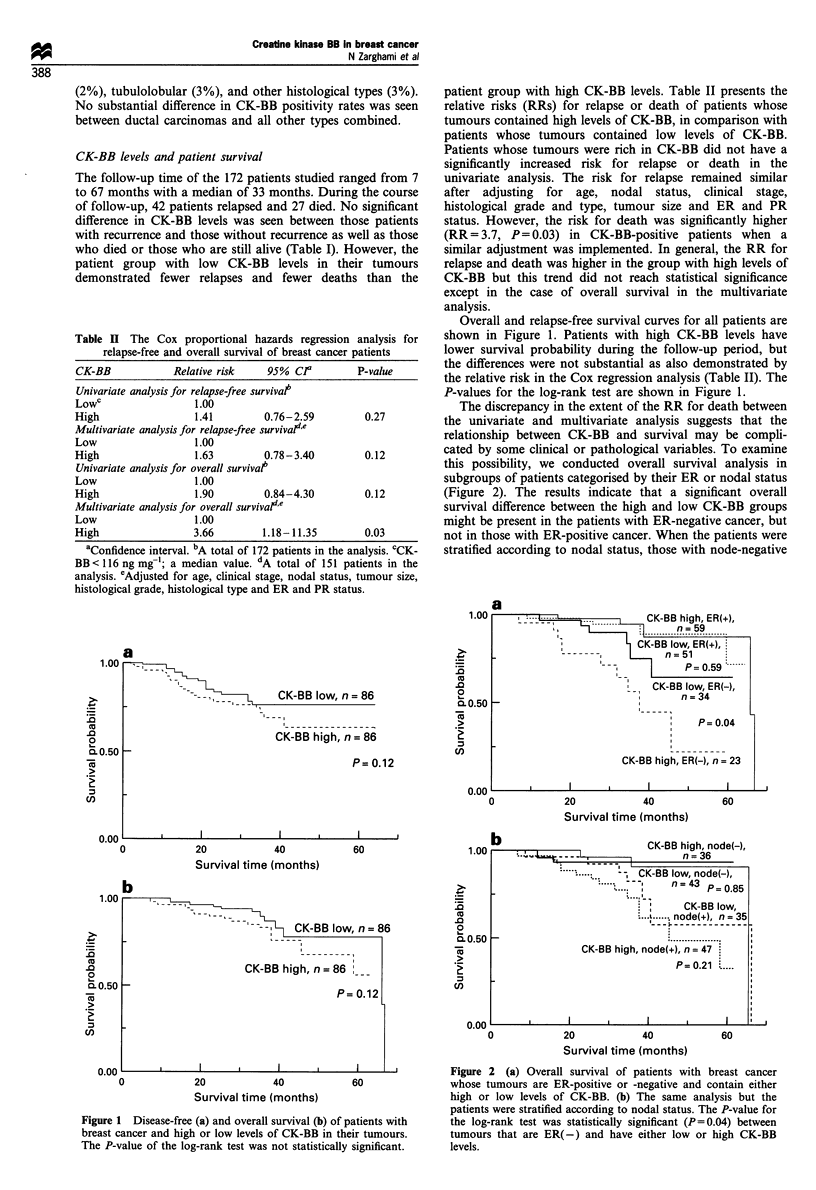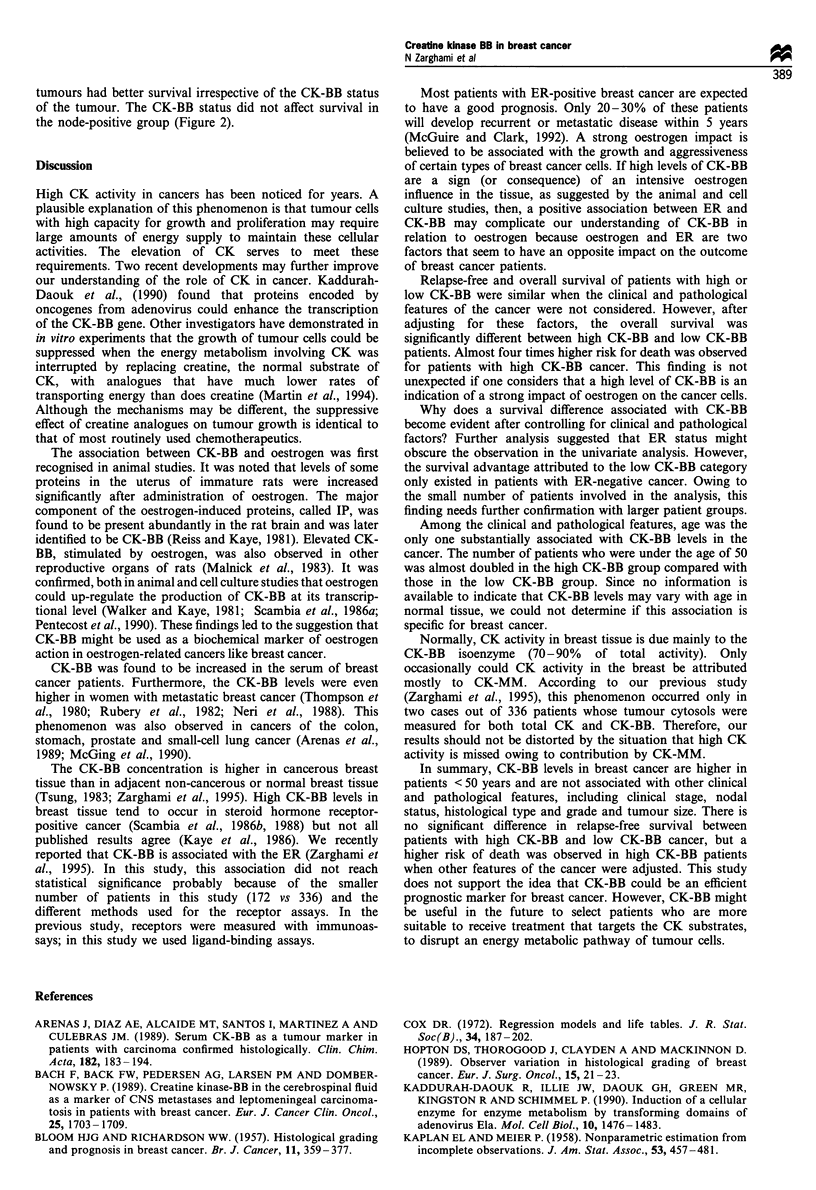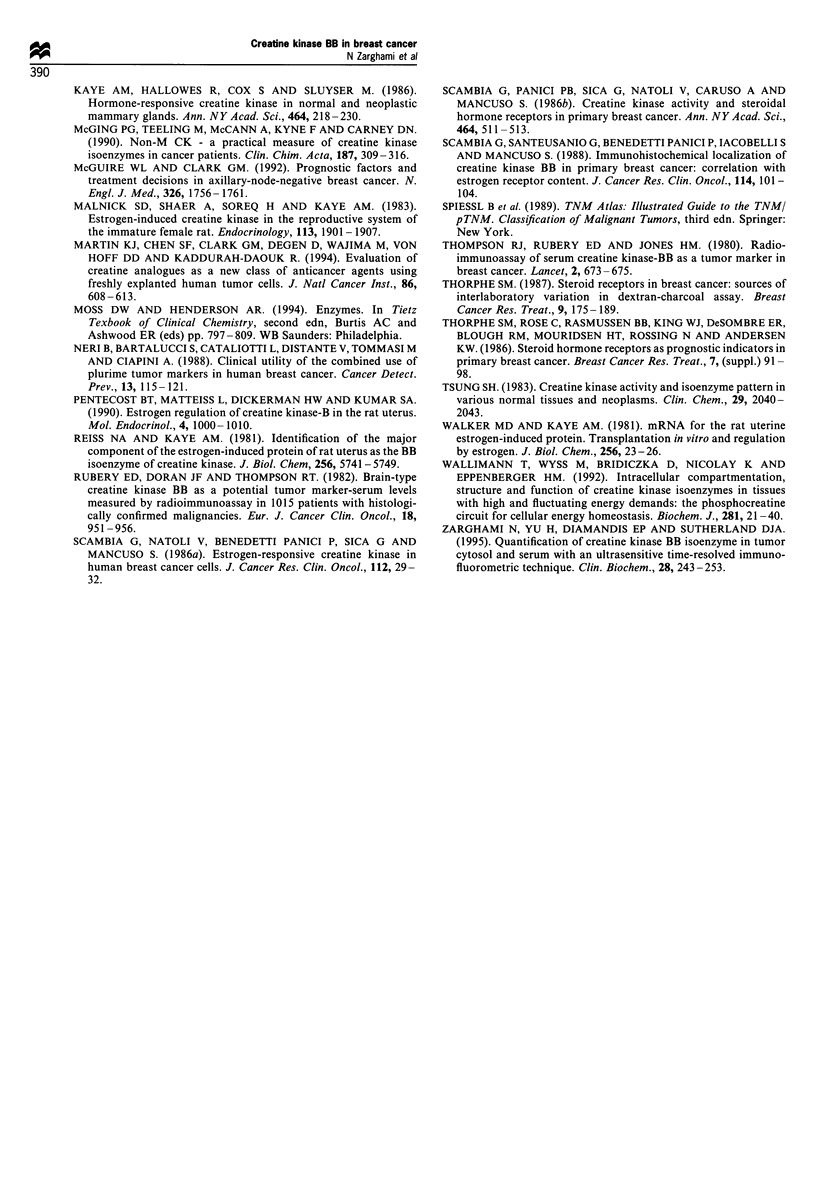# Creatine kinase BB isoenzyme levels in tumour cytosols and survival of breast cancer patients.

**DOI:** 10.1038/bjc.1996.66

**Published:** 1996-02

**Authors:** N. Zarghami, M. Giai, H. Yu, R. Roagna, R. Ponzone, D. Katsaros, P. Sismondi, E. P. Diamandis

**Affiliations:** Department of Pathology and Laboratory Medicine, Mount Sinai Hospital, Toronto, Ontario, Canada.

## Abstract

Creatinine kinase BB (CK-BB) is elevated in many tumours including those of the breast. We have recently described a new, highly sensitive and specific method for measuring CK-BB, based on monoclonal antibodies and time-resolved fluorometry. Using this method, we quantitated CK-BB in 172 breast tumour cytosols and examined the associations between CK-BB and other clinicopathological variables and patient survival. High CK-BB levels were seen more frequently in tumours from patients who were younger (age < 50 years), patients who qualified for chemotherapy and patients with oestrogen receptor-positive tumours. No association was seen between CK-BB and tumour stage, grade, size, histological type or the progesterone receptor. In univariate analysis, the risk of relapse or death was higher in the group with tumours containing high CK-BB levels but the difference did not reach statistical significance. In multivariate analysis, the risk of death was statistically significantly higher in the high-CK-BB group. Analysis of subsets of patients revealed that patients with oestrogen receptor-negative cancer have higher risk of death if their tumours contain high levels of CK-BB. Our data suggest that, in general, CK-BB is associated with more aggressive tumours but its value as a prognostic indicator is limited. CK-BB content of breast tumours may be more useful as an aid in selecting therapy directed at inhibiting this enzyme activity and thus depriving tumour cells of their energy source.


British Journal of Cancer (1996) 73, 386-390
© 1996 Stockton Press All rights reserved 0007-0920/96 $12.00

### Creatine kinase BB isoenzyme levels in tumour cytosols and survival of breast cancer patients

N Zarghami[1], M Giai[2], H Yu[1], R Roagna[2], R Ponzone[2], D Katsaros[2], P Sismondi[2] and EP Diamandis[1]

[1]*Department of Pathology and Laboratory Medicine, Mount Sinai Hospital, 600 University Avenue, Toronto, Ontario M5G 1X5 and Department of Clinical Biochemistry, University of Toronto, 100 College Street, Toronto, Ontario M5G 1L5, Canada;* [2]*Department of Gynecologic Oncology, Institute of Obstetrics and Gynecology, Universtiy of Turin, Italy.*

**Summary** Creatinine kinase BB (CK-BB) is elevated in many tumours including those of the breast. We have recently described a new, highly sensitive and specific method for measuring CK-BB, based on monoclonal antibodies and time-resolved fluorometry. Using this method, we quantitated CK-BB in 172 breast tumour cytosols and examined the associations between CK-BB and other clinicopathological variables and patient survival. High CK-BB levels were seen more frequently in tumours from patients who were younger (age <50 years), patients who qualified for chemotherapy and patients with oestrogen receptor-positive tumours. No association was seen between CK-BB and tumour stage, grade, size, histological type or the progesterone receptor. In univariate analysis, the risk of relapse or death was higher in the group with tumours containing high CK-BB levels but the difference did not reach statistical significance. In multivariate analysis, the risk of death was statistically significantly higher in the high-CK-BB group. Analysis of subsets of patients revealed that patients with oestrogen receptor-negative cancer have higher risk of death if their tumours contain high levels of CK-BB. Our data suggest that, in general, CK-BB is associated with more aggressive tumours but its value as a prognostic indicator is limited. CK-BB content of breast tumours may be more useful as an aid in selecting therapy directed at inhibiting this enzyme activity and thus depriving tumour cells of their energy source.

**Keywords:** breast cancer; creatine kinase BB isoenzyme; prognostic indicators; steroid hormone receptors; enzymes in cancer

Creatine kinase (CK,E.C.2.7.3.2), an enzyme catalysing the reversible phosphorylation between creatine and ATP, plays an important role in energy metabolism. High concentrations of CK may emerge in cells with intensified cellular activities, such as tumour cells, since these cells require high levels of energy. CK is a dimeric molecule with two subunits, M and B. There are three isoenzymes of CK, CK-BB, CK-MB and CK-MM. These CK isoenzymes are present in varying amounts in different tissues. CK-BB is mainly expressed in brain, lung, intestine, bladder, uterus, breast, prostate and placenta. CK-MM predominates in the skeletal muscle and the cardiac muscle is the major location of CK-BB (Moss and Henderson, 1994; Wallimann *et al.*, 1992).

Owing to its close relationship with oestrogen, CK-BB has drawn a lot of attention in breast cancer research. Animal studies have shown enhanced transcription of CK-BB by oestrogen (Walker and Kaye, 1981; Pentecost *et al.*, 1990). Cell culture experiments demonstrated increased CK-BB levels in breast cancer cells after stimulation by oestrogen, (Scambia *et al.*, 1986*a*). Immunohistochemical studies of primary breast cancer tissues indicated a strong staining for CK-BB, which was highly associated with the presence of oestrogen receptor (ER) in the tumour tissue (Scambia *et al.*, 1988). Preliminary clinical investigations found higher serum CK-BB levels in patients with metastatic breast cancer than in patients with cancer in remission (Thompson *et al.*, 1980). Higher levels of CK-BB were also found in the cerebrospinal fluid of patients with breast cancer metastatic to the brain (Bach *et al.*, 1989).

Recently, we developed a new time-resolved immunofluorometric assay for CK-BB. This method is based on two monoclonal anti-CK-BB antibodies and is highly specific and sensitive for the CK-BB isoenzyme (Zarghami *et al.*, 1995). Using this method, we demonstrated that cytosolic CK-BB levels were significantly higher in breast cancer tissue than in normal breast tissue. The presence of CK-BB in tumour tissue was highly associated with the presence of the ER but not with the presence of the progesterone receptor (PR). In the present study we examine whether the CK-BB concentration in breast cancer cytosols is associated with other clinical and pathological features of breast cancer as well as the survival of breast cancer patients.

Correspondence: E P Diamandis, Department of Pathology and Laboratory Medicine, Mount Sinai Hospital, 600 University Avenue, Toronto, Ontario M5G 1X5, Canada
Received 4 April 1995; revised 1 August 1995; accepted 31 August 1995

### Materials and methods

### *Patients with breast cancer*

Tumour tissue was collected from 172 primary breast cancer patients aged between 25 and 91 years (median age 56 years). These patients were diagnosed and/or treated in the Department of Gynecologic Oncology, University of Turin, between January 1988 and December 1991. They represented 70% of all new breast cancer cases seen at the department during that time period. They were consecutive cases collected under the condition that sufficient tissue remained after pathological examination and receptor analysis. Patients with bilateral lesions, Paget's disease of the breast, or disseminated disease at the time of diagnosis or within 2 months after surgery, and patients who received only palliative treatment were excluded from the study.

All patients in this study were treated with modified mastectomy or conservative surgery plus post-surgical irradiation of the breast. Axillary lymph nodes were examined by pathologists for 163 patients; the mean number of nodes examined was $15 \pm 6$ (one standard deviation). Eleven patients did not undergo node dissection because they were older than 75 years. The patients were followed-up clinically every 3 months for the first 2 years after surgery, every 6 months for the next 3 years and then annually.

Patients were staged according to the International Union Against Cancer post-surgical tumour–node–metastasis (pTNM) classification (Spiessl et al., 1989). Tumour specimens were histologically graded and typed based on criteria previously described (Bloom and Richardson, 1957; Hopton et al., 1989). The tumour size, recorded as the maximum diameter of the fresh mastectomy specimen ranged from 0.7 to 6 cm with a median size of 2.4 cm.

Adjuvant tamoxifen treatment was given to node-positive post-menopausal patients and adjuvant chemotherapy CMF (cyclophosphamide–methotrexate–5–fluorouracil, intravenously) was given to node-positive premenopausal patients. If there was no indication of relapse, node-negative patients did not receive adjuvant treatment after surgery. None of the patients received adjuvant therapy before surgery.

*Cytosol extraction*

Tissue specimens were snap frozen in liquid nitrogen immediately after surgical removal and were stored at −80°C until cytosol extraction. About 0.2 g of tumour tissue was pulverised manually to a fine powder at −80°C, and the cells were lysed for 30 min on ice with 2 ml of lysis buffer (50 mmol $l^{-1}$ Tris buffer, pH 8.0, containing 150 mmol sodium chloride, 5 mmol EDTA, 10 g Nonidet NP-40 surfactant and 1 mmol phenylmethylsulphonyl fluoride $l^{-1}$). The lysate was centrifuged at 15 000 ***g*** at 4°C for 30 min and the supernatant was collected for measurement of CK-BB and total protein.

*Measurement of CK-BB and other markers*

CK-BB concentration in breast cancer cytosols was measured in duplicate with a time-resolved immunofluorometric assay described in detail elsewhere (Zarghami et al., 1995). The assay has a limit of detection around 0.002 ng $ml^{-1}$ and a measurement range to 10 ng $ml^{-1}$; the within-run coefficient of variation (CV) is 4–9% and the between run CV is 6–12%. The assay has no cross-reactivity with CK-MM and a minimal cross-reactivity (3%) with CK-BB. The assay uses two monoclonal antibodies (OEM Concepts, Toms River, NJ, USA) one of which was coated to white microtitre wells and the other one was biotinylated. For detection, we used streptavidin conjugated to alkaline phosphatase (ALP). The ALP substrate was 5'-fluorosalicylphosphate which, upon hydrolysis, forms highly fluorescent complexes with $Tb^{3+}$ and EDTA.

Total protein concentration in the tumour cytosols was measured using a commercial kit based on the bicinchoninic acid (BCA) method (Pierce Chemical, Rockford, IL, USA). ERs and PRs were measured with the dextran-coated charcoal (DCC) method (Thorphe et al., 1986, 1987) and the results were grouped into receptor-positive and -negative categories based on a cut-off value of 10 fmol $mg^{-1}$ total protein.

*Statistical analysis*

CK-BB values were categorised into two groups, high and low levels of CK-BB, using the median as a cut-off point. Relationships between CK-BB levels and other clinicopathological variables, including patient's age, clinical stage, histological grade and type, nodal status, tumour size, ER, PR and adjuvant treatment, were analysed using the contingency table and chi-square test. Associations between CK-BB levels and patient survival (relapse-free survival and overall survival) were evaluated using both the Kaplan–Meier survival analysis method (Kaplan and Meier, 1958) and the Cox proportional hazards regression model (Cox, 1972). Computer software SAS (SAS Institute, Cary, NC, USA) and EGRET (Statistics and Epidemiology Research Corporation, Seattle, WA, USA) were employed.

### Results

*Association between CK-BB and other clinicopathological variables*

A total of 172 tumour extracts were analysed for CK-BB. CK-BB levels in tumour cytosols varied widely from 5 to 2857 ng of CK-BB per mg of total protein with a median of 116 ng $mg^{-1}$. Table I describes the association between CK-BB status and other clinical and pathological variables. Cancers with high levels of CK-BB were found more frequently in younger patients (<50 years) than in older patients (42% *vs* 22%). The difference was statistically significant ($P<0.01$).

Patients with high levels of CK-BB in their cancer were more likely to receive chemotherapy or chemotherapy plus tamoxifen than did those with low CK-BB in their cancer (28% *vs* 12%, $P=0.03$). This observation suggests that cancers with high CK-BB were more aggressive and, as a result, more patients from this group qualified to receive chemotherapy. However, when we examined the relationship between CK-BB status and nodal status, a trend was seen associating high CK-BB with positive nodal status only ($P=0.14$). Clinical stage, histological grade or tumour size, did not significantly associate with the CK-BB level in the breast tumours ($P>0.23$ in all cases).

High CK-BB levels were associated with presence of the ER but the difference did not reach statistical significance ($P=0.10$). The PR does not seem to be associated with CK-BB ($P=0.25$), in accordance with previous results (Zarghami et al., 1995).

Most patients studied (70%) had ductal carcinomas. The rest of the histological types observed were lobular (13%), lobular *in situ* (2%), medullary (5%), papillary (2%), tubular

**Table I** Associations between CK-BB levels in breast tumour cytosols and other clinicopathological variables

| *Variable* | *CK-BB, high[a] Patients (%)* | *CK-BB, low Patients (%)* | P-*value* |
|---|---|---|---|
| *Age (years)* | | | |
| <50 | 36 (41.9) | 19 (22.1) | |
| ⩾50 | 50 (58.1) | 67 (77.9) | <0.01 |
| *Clinical stage* | | | |
| I | 41 (47.7) | 36 (42.4) | |
| II | 38 (44.2) | 43 (50.6) | |
| II–IV | 7 (8.1) | 6 (7.0) | 0.70 |
| *Nodal status* | | | |
| Negative | 36 (43.4) | 43 (55.1) | |
| Positive | 47 (56.6) | 35 (44.9) | 0.14 |
| *Tumour size (cm)* | | | |
| <1.5 | 14 (16.7) | 10 (11.9) | |
| ⩾1.5 | 70 (83.3) | 74 (88.1) | 0.38 |
| *Histological type* | | | |
| Ductal | 62 (72.1) | 58 (67.4) | |
| Others | 24 (27.9) | 28 (32.6) | 0.51 |
| *Histological grade* | | | |
| I | 28 (32.6) | 39 (45.4) | |
| II | 40 (46.5) | 32 (37.2) | |
| III | 18 (20.9) | 15 (17.4) | 0.23 |
| *ER status[b]* | | | |
| Negative | 23 (28.1) | 34 (40.0) | |
| Positive | 59 (71.9) | 51 (60.0) | 0.10 |
| *PR status[b]* | | | |
| Negative | 30 (37.0) | 39 (45.9) | |
| Positive | 51 (63.0) | 46 (54.1) | 0.25 |
| *Adjuvant treatment* | | | |
| None | 33 (38.4) | 42 (48.8) | |
| Tamoxifen | 29 (33.7) | 34 (39.5) | |
| Chemo ± tamoxifen | 24 (27.9) | 10 (11.6) | 0.03 |
| *Relapse* | | | |
| No | 62 (72.1) | 68 (79.1) | |
| Yes | 24 (27.9) | 18 (20.9) | 0.29 |
| *Death* | | | |
| No | 70 (81.4) | 75 (87.2) | |
| Yes | 16 (18.6) | 11 (12.8) | 0.30 |

[a]CK-BB ⩾ 116 ng $mg^{-1}$; a median value. [b]Cut-off of 10 fmol $mg^{-1}$.

(2%), tubulolobular (3%), and other histological types (3%). No substantial difference in CK-BB positivity rates was seen between ductal carcinomas and all other types combined.

### *CK-BB levels and patient survival*

The follow-up time of the 172 patients studied ranged from 7 to 67 months with a median of 33 months. During the course of follow-up, 42 patients relapsed and 27 died. No significant difference in CK-BB levels was seen between those patients with recurrence and those without recurrence as well as those who died or those who are still alive (Table I). However, the patient group with low CK-BB levels in their tumours demonstrated fewer relapses and fewer deaths than the patient group with high CK-BB levels. Table II presents the relative risks (RRs) for relapse or death of patients whose tumours contained high levels of CK-BB, in comparison with patients whose tumours contained low levels of CK-BB. Patients whose tumours were rich in CK-BB did not have a significantly increased risk for relapse or death in the univariate analysis. The risk for relapse remained similar after adjusting for age, nodal status, clinical stage, histological grade and type, tumour size and ER and PR status. However, the risk for death was significantly higher (RR = 3.7, $P = 0.03$) in CK-BB-positive patients when a similar adjustment was implemented. In general, the RR for relapse and death was higher in the group with high levels of CK-BB but this trend did not reach statistical significance except in the case of overall survival in the multivariate analysis.

Overall and relapse-free survival curves for all patients are shown in Figure 1. Patients with high CK-BB levels have lower survival probability during the follow-up period, but the differences were not substantial as also demonstrated by the relative risk in the Cox regression analysis (Table II). The $P$-values for the log-rank test are shown in Figure 1.

The discrepancy in the extent of the RR for death between the univariate and multivariate analysis suggests that the relationship between CK-BB and survival may be complicated by some clinical or pathological variables. To examine this possibility, we conducted overall survival analysis in subgroups of patients categorised by their ER or nodal status (Figure 2). The results indicate that a significant overall survival difference between the high and low CK-BB groups might be present in the patients with ER-negative cancer, but not in those with ER-positive cancer. When the patients were stratified according to nodal status, those with node-negative

**Table II** The Cox proportional hazards regression analysis for relapse-free and overall survival of breast cancer patients

| *CK-BB* | *Relative risk* | *95% CI*[a] | P-*value* |
|---|---|---|---|
| *Univariate analysis for relapse-free survival*[b] | | | |
| Low[c] | 1.00 | | |
| High | 1.41 | 0.76–2.59 | 0.27 |
| *Multivariate analysis for relapse-free survival*[d,e] | | | |
| Low | 1.00 | | |
| High | 1.63 | 0.78–3.40 | 0.12 |
| *Univariate analysis for overall survival*[b] | | | |
| Low | 1.00 | | |
| High | 1.90 | 0.84–4.30 | 0.12 |
| *Multivariate analysis for overall survival*[d,e] | | | |
| Low | 1.00 | | |
| High | 3.66 | 1.18–11.35 | 0.03 |

[a]Confidence interval. [b]A total of 172 patients in the analysis. [c]CK-BB < 116 ng $mg^{-1}$; a median value. [d]A total of 151 patients in the analysis. [e]Adjusted for age, clinical stage, nodal status, tumour size, histological grade, histological type and ER and PR status.

**Figure 1** Disease-free (**a**) and overall survival (**b**) of patients with breast cancer and high or low levels of CK-BB in their tumours. The $P$-value of the log-rank test was not statistically significant.

**Figure 2** (**a**) Overall survival of patients with breast cancer whose tumours are ER-positive or -negative and contain either high or low levels of CK-BB. (**b**) The same analysis but the patients were stratified according to nodal status. The $P$-value for the log-rank test was statistically significant ($P = 0.04$) between tumours that are ER(−) and have either low or high CK-BB levels.

tumours had better survival irrespective of the CK-BB status of the tumour. The CK-BB status did not affect survival in the node-positive group (Figure 2).

### Discussion

High CK activity in cancers has been noticed for years. A plausible explanation of this phenomenon is that tumour cells with high capacity for growth and proliferation may require large amounts of energy supply to maintain these cellular activities. The elevation of CK serves to meet these requirements. Two recent developments may further improve our understanding of the role of CK in cancer. Kaddurah-Daouk *et al.*, (1990) found that proteins encoded by oncogenes from adenovirus could enhance the transcription of the CK-BB gene. Other investigators have demonstrated in *in vitro* experiments that the growth of tumour cells could be suppressed when the energy metabolism involving CK was interrupted by replacing creatine, the normal substrate of CK, with analogues that have much lower rates of transporting energy than does creatine (Martin *et al.*, 1994). Although the mechanisms may be different, the suppressive effect of creatine analogues on tumour growth is identical to that of most routinely used chemotherapeutics.

The association between CK-BB and oestrogen was first recognised in animal studies. It was noted that levels of some proteins in the uterus of immature rats were increased significantly after administration of oestrogen. The major component of the oestrogen-induced proteins, called IP, was found to be present abundantly in the rat brain and was later identified to be CK-BB (Reiss and Kaye, 1981). Elevated CK-BB, stimulated by oestrogen, was also observed in other reproductive organs of rats (Malnick *et al.*, 1983). It was confirmed, both in animal and cell culture studies that oestrogen could up-regulate the production of CK-BB at its transcriptional level (Walker and Kaye, 1981; Scambia *et al.*, 1986*a*; Pentecost *et al.*, 1990). These findings led to the suggestion that CK-BB might be used as a biochemical marker of oestrogen action in oestrogen-related cancers like breast cancer.

CK-BB was found to be increased in the serum of breast cancer patients. Furthermore, the CK-BB levels were even higher in women with metastatic breast cancer (Thompson *et al.*, 1980; Rubery *et al.*, 1982; Neri *et al.*, 1988). This phenomenon was also observed in cancers of the colon, stomach, prostate and small-cell lung cancer (Arenas *et al.*, 1989; McGing *et al.*, 1990).

The CK-BB concentration is higher in cancerous breast tissue than in adjacent non-cancerous or normal breast tissue (Tsung, 1983; Zarghami *et al.*, 1995). High CK-BB levels in breast tissue tend to occur in steroid hormone receptor-positive cancer (Scambia *et al.*, 1986*b*, 1988) but not all published results agree (Kaye *et al.*, 1986). We recently reported that CK-BB is associated with the ER (Zarghami *et al.*, 1995). In this study, this association did not reach statistical significance probably because of the smaller number of patients in this study (172 *vs* 336) and the different methods used for the receptor assays. In the previous study, receptors were measured with immunoassays; in this study we used ligand-binding assays.

Most patients with ER-positive breast cancer are expected to have a good prognosis. Only 20–30% of these patients will develop recurrent or metastatic disease within 5 years (McGuire and Clark, 1992). A strong oestrogen impact is believed to be associated with the growth and aggressiveness of certain types of breast cancer cells. If high levels of CK-BB are a sign (or consequence) of an intensive oestrogen influence in the tissue, as suggested by the animal and cell culture studies, then, a positive association between ER and CK-BB may complicate our understanding of CK-BB in relation to oestrogen because oestrogen and ER are two factors that seem to have an opposite impact on the outcome of breast cancer patients.

Relapse-free and overall survival of patients with high or low CK-BB were similar when the clinical and pathological features of the cancer were not considered. However, after adjusting for these factors, the overall survival was significantly different between high CK-BB and low CK-BB patients. Almost four times higher risk for death was observed for patients with high CK-BB cancer. This finding is not unexpected if one considers that a high level of CK-BB is an indication of a strong impact of oestrogen on the cancer cells.

Why does a survival difference associated with CK-BB become evident after controlling for clinical and pathological factors? Further analysis suggested that ER status might obscure the observation in the univariate analysis. However, the survival advantage attributed to the low CK-BB category only existed in patients with ER-negative cancer. Owing to the small number of patients involved in the analysis, this finding needs further confirmation with larger patient groups.

Among the clinical and pathological features, age was the only one substantially associated with CK-BB levels in the cancer. The number of patients who were under the age of 50 was almost doubled in the high CK-BB group compared with those in the low CK-BB group. Since no information is available to indicate that CK-BB levels may vary with age in normal tissue, we could not determine if this association is specific for breast cancer.

Normally, CK activity in breast tissue is due mainly to the CK-BB isoenzyme (70–90% of total activity). Only occasionally could CK activity in the breast be attributed mostly to CK-MM. According to our previous study (Zarghami *et al.*, 1995), this phenomenon occurred only in two cases out of 336 patients whose tumour cytosols were measured for both total CK and CK-BB. Therefore, our results should not be distorted by the situation that high CK activity is missed owing to contribution by CK-MM.

In summary, CK-BB levels in breast cancer are higher in patients $<50$ years and are not associated with other clinical and pathological features, including clinical stage, nodal status, histological type and grade and tumour size. There is no significant difference in relapse-free survival between patients with high CK-BB and low CK-BB cancer, but a higher risk of death was observed in high CK-BB patients when other features of the cancer were adjusted. This study does not support the idea that CK-BB could be an efficient prognostic marker for breast cancer. However, CK-BB might be useful in the future to select patients who are more suitable to receive treatment that targets the CK substrates, to disrupt an energy metabolic pathway of tumour cells.

### References

ARENAS J, DIAZ AE, ALCAIDE MT, SANTOS I, MARTINEZ A AND CULEBRAS JM. (1989). Serum CK-BB as a tumour marker in patients with carcinoma confirmed histologically. *Clin. Chim. Acta*, **182**, 183–194.

BACH F, BACK FW, PEDERSEN AG, LARSEN PM AND DOMBERNOWSKY P. (1989). Creatine kinase-BB in the cerebrospinal fluid as a marker of CNS metastases and leptomeningeal carcinomatosis in patients with breast cancer. *Eur. J. Cancer Clin. Oncol.*, **25**, 1703–1709.

BLOOM HJG AND RICHARDSON WW. (1957). Histological grading and prognosis in breast cancer. *Br. J. Cancer*, **11**, 359–377.

COX DR. (1972). Regression models and life tables. *J. R. Stat. Soc(B).*, **34**, 187–202.

HOPTON DS, THOROGOOD J, CLAYDEN A AND MACKINNON D. (1989). Observer variation in histological grading of breast cancer. *Eur. J. Surg. Oncol.*, **15**, 21–23.

KADDURAH-DAOUK R, ILLIE JW, DAOUK GH, GREEN MR, KINGSTON R AND SCHIMMEL P. (1990). Induction of a cellular enzyme for enzyme metabolism by transforming domains of adenovirus Ela. *Mol. Cell Biol.*, **10**, 1476–1483.

KAPLAN EL AND MEIER P. (1958). Nonparametric estimation from incomplete observations. *J. Am. Stat. Assoc.*, **53**, 457–481.

KAYE AM, HALLOWES R, COX S AND SLUYSER M. (1986). Hormone-responsive creatine kinase in normal and neoplastic mammary glands. *Ann. NY Acad. Sci.*, **464**, 218–230.

McGING PG, TEELING M, McCANN A, KYNE F AND CARNEY DN. (1990). Non-M CK - a practical measure of creatine kinase isoenzymes in cancer patients. *Clin. Chim. Acta*, **187**, 309–316.

McGUIRE WL AND CLARK GM. (1992). Prognostic factors and treatment decisions in axillary-node-negative breast cancer. *N. Engl. J. Med.*, **326**, 1756–1761.

MALNICK SD, SHAER A, SOREQ H AND KAYE AM. (1983). Estrogen-induced creatine kinase in the reproductive system of the immature female rat. *Endocrinology*, **113**, 1901–1907.

MARTIN KJ, CHEN SF, CLARK GM, DEGEN D, WAJIMA M, VON HOFF DD AND KADDURAH-DAOUK R. (1994). Evaluation of creatine analogues as a new class of anticancer agents using freshly explanted human tumor cells. *J. Natl Cancer Inst.*, **86**, 608–613.

MOSS DW AND HENDERSON AR. (1994). Enzymes. In *Tietz Texbook of Clinical Chemistry*, second edn, Burtis AC and Ashwood ER (eds) pp. 797–809. WB Saunders: Philadelphia.

NERI B, BARTALUCCI S, CATALIOTTI L, DISTANTE V, TOMMASI M AND CIAPINI A. (1988). Clinical utility of the combined use of plurime tumor markers in human breast cancer. *Cancer Detect. Prev.*, **13**, 115–121.

PENTECOST BT, MATTEISS L, DICKERMAN HW AND KUMAR SA. (1990). Estrogen regulation of creatine kinase-B in the rat uterus. *Mol. Endocrinol.*, **4**, 1000–1010.

REISS NA AND KAYE AM. (1981). Identification of the major component of the estrogen-induced protein of rat uterus as the BB isoenzyme of creatine kinase. *J. Biol. Chem*, **256**, 5741–5749.

RUBERY ED, DORAN JF AND THOMPSON RT. (1982). Brain-type creatine kinase BB as a potential tumor marker-serum levels measured by radioimmunoassay in 1015 patients with histologically confirmed malignancies. *Eur. J. Cancer Clin. Oncol.*, **18**, 951–956.

SCAMBIA G, NATOLI V, BENEDETTI PANICI P, SICA G AND MANCUSO S. (1986*a*). Estrogen-responsive creatine kinase in human breast cancer cells. *J. Cancer Res. Clin. Oncol.*, **112**, 29–32.

SCAMBIA G, PANICI PB, SICA G, NATOLI V, CARUSO A AND MANCUSO S. (1986*b*). Creatine kinase activity and steroidal hormone receptors in primary breast cancer. *Ann. NY Acad. Sci.*, **464**, 511–513.

SCAMBIA G, SANTEUSANIO G, BENEDETTI PANICI P, IACOBELLI S AND MANCUSO S. (1988). Immunohistochemical localization of creatine kinase BB in primary breast cancer: correlation with estrogen receptor content. *J. Cancer Res. Clin. Oncol.*, **114**, 101–104.

SPIESSL B *et al.* (1989). *TNM Atlas: Illustrated Guide to the TNM/pTNM. Classification of Malignant Tumors*, third edn. Springer: New York.

THOMPSON RJ, RUBERY ED AND JONES HM. (1980). Radioimmunoassay of serum creatine kinase-BB as a tumor marker in breast cancer. *Lancet*, **2**, 673–675.

THORPHE SM. (1987). Steroid receptors in breast cancer: sources of interlaboratory variation in dextran-charcoal assay. *Breast Cancer Res. Treat.*, **9**, 175–189.

THORPHE SM, ROSE C, RASMUSSEN BB, KING WJ, DeSOMBRE ER, BLOUGH RM, MOURIDSEN HT, ROSSING N AND ANDERSEN KW. (1986). Steroid hormone receptors as prognostic indicators in primary breast cancer. *Breast Cancer Res. Treat.*, **7**, (suppl.) 91–98.

TSUNG SH. (1983). Creatine kinase activity and isoenzyme pattern in various normal tissues and neoplasms. *Clin. Chem.*, **29**, 2040–2043.

WALKER MD AND KAYE AM. (1981). mRNA for the rat uterine estrogen-induced protein. Transplantation *in vitro* and regulation by estrogen. *J. Biol. Chem.*, **256**, 23–26.

WALLIMANN T, WYSS M, BRIDICZKA D, NICOLAY K AND EPPENBERGER HM. (1992). Intracellular compartmentation, structure and function of creatine kinase isoenzymes in tissues with high and fluctuating energy demands: the phosphocreatine circuit for cellular energy homeostasis. *Biochem. J.*, **281**, 21–40.

ZARGHAMI N, YU H, DIAMANDIS EP AND SUTHERLAND DJA. (1995). Quantification of creatine kinase BB isoenzyme in tumor cytosol and serum with an ultrasensitive time-resolved immunofluorometric technique. *Clin. Biochem.*, **28**, 243–253.